# A Rare Case of Brucellosis With Spontaneous Splenic Rupture Presenting as an Acute Abdomen

**DOI:** 10.7759/cureus.28753

**Published:** 2022-09-03

**Authors:** Farhana Siraj, Amrit Dhar, Afshan Shabir, Suhail Mantoo, Umar H Khan

**Affiliations:** 1 Internal and Pulmonary Medicine, Sher-i-Kashmir Institute of Medical Sciences, Srinagar, IND

**Keywords:** atraumatic, spontaneous, hemoperitoneum, splenic rupture, brucellosis

## Abstract

Brucellosis is a common zoonotic infection worldwide caused by the bacterial species Brucella. It has a wide range of presentations from asymptomatic infection to multisystem involvement. Splenomegaly is seen in around 30-60% of cases, however, atraumatic spontaneous splenic rupture is extremely rare. We present a case of a 45-year-old man who presented with acute left upper quadrant pain and fever of five days duration without a history of antecedent trauma. He was hemodynamically stable with examination revealing left upper quadrant tender palpable mass. Ultrasonography followed by computed tomography revealed subcapsular hematoma with perisplenic and perihepatic free fluid. Viral markers (hepatitis B and C, cytomegalovirus {CMV}, Epstein-Barr virus {EBV}, HIV, and dengue) were negative. The autoimmune profile was negative. Brucella serum agglutination test was positive (1: 640) and blood cultures grew *Brucella melitensis*. He was managed conservatively for splenic hematoma and received one unit blood transfusion and treatment with combination of antibiotics (rifampicin and doxycycline) for brucella for six weeks. On follow-up, the patient reported no further complications. Spontaneous splenic rupture is a clinical rarity and should be considered in patients presenting with acute abdomen and suspected infective, neoplastic, and inflammatory pathology. Spontaneous splenic rupture in acute brucellosis requires prompt clinical recognition and immediate anti-Brucella therapy to prevent the catastrophic progression.

## Introduction

Brucellosis, a zoonotic infection seen worldwide, is caused by a bacterial species Brucella, a Gram-negative bacillus. It has a wide range of presentations, ranging from asymptomatic infection, and non-specific symptoms (fever, fatigue, sweating) to having a multisystem involvement. Almost all systems are involved including the spleen [1,2].

Gastrointestinal manifestations of brucellosis are seen in up to 40% of cases, these include anorexia, abdominal pain, constipation, and weight loss being the commonest. Complications of brucellosis include Brucella hepatitis with abscess formation, splenic abscess, spontaneous rupture of the spleen, cholecystitis, intestinal obstruction or perforation, and pancreatitis which can manifest as acute abdomen [3].

Splenic enlargement in brucellosis is reported in around 30-60% of cases [4]. Spontaneous splenic rupture (SSR) is an uncommon clinical entity, reported in infectious, neoplastic, autoimmune, or hematological diseases. Infectious causes account majorly for SSR cases, the underlying etiology varying according to the geographical location. Whereas malaria and tuberculosis are most common in Africa and Asia; Babesia as a cause has been found in the USA [5,6].

To date, only five cases of SSR as a complication of brucellosis have been reported worldwide [7-11]. Herein, we present another case of brucellosis with spontaneous splenic rupture. Clinicians should be vigilant for splenic rupture in any patient with brucellosis and acute abdomen.

## Case presentation

A 45-year-old male, a farmer by occupation, with no underlying co-morbid illness presented to the emergency department with acute abdominal pain in the left hypochondrium for a few hours duration. He had a history of high-grade fever (maximum of 103°F) for the last five days. Being a farmer, the patient had exposure to cattle. However, there was no history of antecedent trauma in the last two weeks, and no history of any other family member being ill. On clinical examination, there was pallor and tachycardia (114 beats/min) and blood pressure was 110/70 mmHg. He was anxious in appearance and was wincing with abdominal pain. Abdominal examination revealed mild distension with a palpable mass of 4-5 cm below the left costal margin, in addition, there was tenderness (Ballance sign) and rebound in the left hypochondrium.

Baseline investigations as seen in Table 1 show normocytic anemia. Peripheral blood did not reveal any atypical cells or blasts. Erythrocyte sedimentation rate (ESR) was raised. Serum biochemistry (renal function, liver function) was normal, and serum amylase was mildly raised, meanwhile, coagulation was normal.

**Table 1:** Baseline laboratory investigations of the patient. MCV: mean corpuscular volume; TLC: total leucocyte count; ESR: erythrocyte sedimentation rate; ALT: alanine aminotransferase; INR: international normalized ratio

Variable	Patient value	Reference value
Hemoglobin (g/dL)	8.8	12-16
MCV (fL)	88	80-100
TLC (mm^3^)	7800	4000-11,000
Platelets (10^3^/mm^3^)	145	150-400
ESR (mm/h)	88	0-15
Blood urea (mg/dL)	38	10-45
Serum creatinine (mg/dL)	0.88	0.5-1.2
Serum bilirubin (mg/dL)	1.01	0.3-1.5
Serum ALT (IU/L)	37	0-45
Serum albumin (g/dL)	3.88	3.5-5.5
Serum amylase (IU/L)	160	40-140
INR	1.04	0.8-1.1

**Figure 1:**
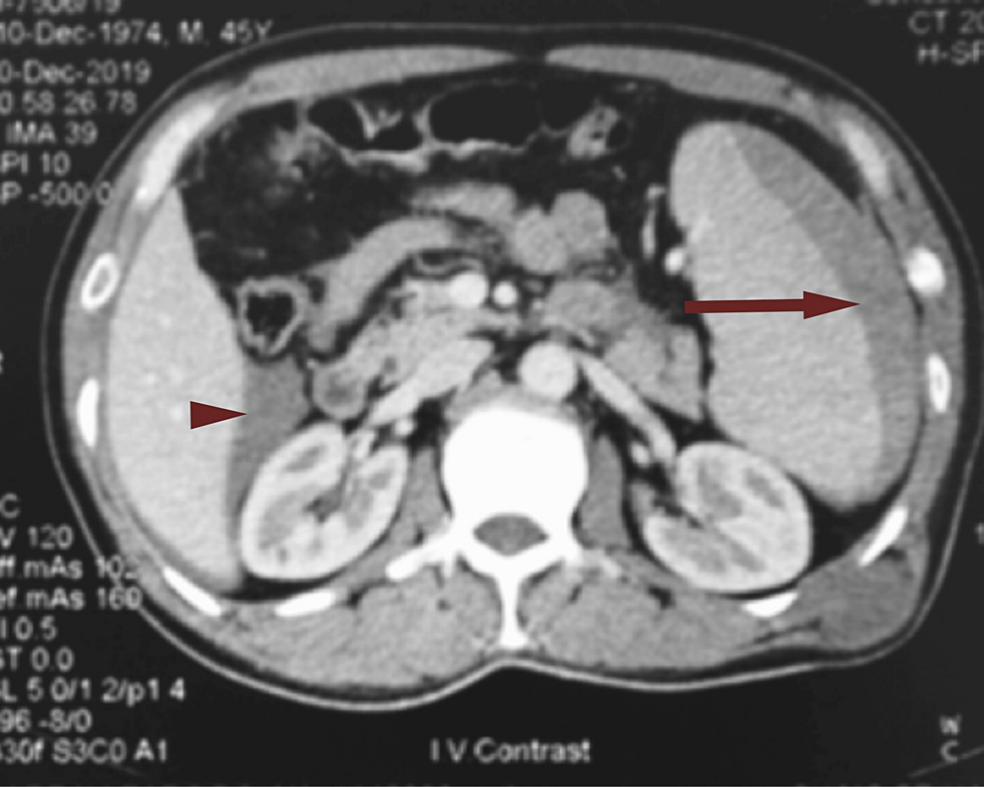
Contrast CT abdomen suggestive of a large subcapsular splenic hematoma (arrow) with moderate free fluid in perihepatic region (arrowhead).

Ultrasonography showed splenomegaly with free fluid in the abdomen. Contrast-enhanced computed tomography (CECT) of the abdomen and pelvis was sought, splenomegaly with a large subcapsular splenic hematoma with moderate free fluid in perisplenic, perihepatic, and pelvic regions suggestive of splenic rupture with hemoperitoneum was found (Figure 1). Diagnostic abdominal paracentesis revealed a bloody tap with 60 cells (80% neutrophils, 20% lymphocytes) and an entire field of red cells.

Viral serology for hepatitis B and C, Ebstein-Barr virus (EBV), cytomegalovirus (CMV), and HIV were negative. Malaria by card test and dengue serology were negative. The autoimmune profile (anti-nuclear antibody, rheumatoid factor, anti-cyclic citrullinated peptide, perinuclear anti-neutrophil cytoplasmic antibodies {p-ANCA}, cytoplasmic anti-neutrophil cytoplasmic antibodies {c-ANCA}) was negative. Blood culture grew *Brucella melitensis* with serum agglutination test revealing titers of greater than 1:640 for *Brucella melitensis *and higher dilution revealing titers of 1:2560. The echocardiographic examination was normal. Conservative management for splenic rupture was considered after consultation with the surgical team. He received intravenous fluids, one unit blood transfusion, and daily clinical and biochemical monitoring was done. Hemoglobin had risen to 10.1 g/dL post blood transfusion, and no further drop in hemoglobin during the course of hospitalization was seen. He was started on triple therapy for brucellosis with rifampicin, doxycycline for six weeks, and streptomycin initially for a total duration of three weeks. Repeat ultrasonography at six weeks revealed resolution of hemoperitoneum and subcapsular splenic hematoma. On follow-up as an outpatient, no complications or sequelae developed.

## Discussion

Rupture of the spleen is a commonly seen complication following blunt abdominal trauma. An atraumatic rupture in a diseased spleen is seen less frequently but has been widely reported in the literature [12]. In contrast, in absence of these two risk factors, spontaneous splenic rupture (SSR) is rarely reported and is poorly defined [5].

The first case of SSR was described in 1874 by Atkinson, on post-mortem analysis [13]. A systemic review in 2012 evaluated 613 cases of splenic rupture without risk factors or previously diagnosed diseases and found that atraumatic splenic rupture can be an initial presenting complaint of a previously undiagnosed condition [5]. This was seen in our case, where brucellosis was the underlying cause for splenic rupture. Renzulli et al. corroborated these findings and found that in 51.2% of all cases of atraumatic splenic rupture, the underlying cause was elicited only after presenting to the hospital [12].

SSR has been widely reported in numerous infective, neoplastic, inflammatory, rheumatological, and hematological diseases. Malaria has been implicated in most cases of all the infective causes, followed by infectious mononucleosis (EBV), CMV, HIV, Salmonella, brucellosis, babesiosis, and tuberculosis [5,6].

Brucellosis as a cause of SSR is very rare and ours is the sixth case reported in the literature. Table 2 depicts cases reported in the literature and their varying presentation. Of the total five cases, three have been reported from Turkey, and the other two are from Spain. Ours is the first case to be reported from the Asian sub-continent, however, we believe that cases are either under-reported or misdiagnosed. In two of the cases, the patient presented with hemodynamic instability (tachycardia, hypotension) along with dizziness and abdominal pain. Massive hemoperitoneum was found which necessitated exploration and splenectomy in one of the cases. In another case, the patient had non-specific presenting symptoms and on evaluation was found to have underlying brucellosis, thus, highlighting the spectrum of disease presentation in acute brucellosis. Our patient had a mixed presentation of the above, where constitutional symptoms and acute abdominal pain co-existed. He was found to have splenic rupture with moderate hemoperitoneum.

**Table 2:** The clinical presentation and outcomes of various cases reported in literature. CECT: contrast-enhanced computed tomography; N/A: abstract not available

Case	Authors	Country/year of publication	Age/sex	Clinical presentation	Treatment received and outcome
1	Yagmurkaya et al.	Turkey/2021	52/M	Pain abdomen, vomiting, vertigo, hypotension CECT suggestive of ruptured spleen and widespread hemorrhagic fluid in abdomen.	Emergency exploration and splenectomy were done. Medical management continued. Duration N/A. No post-operative complication.
2	Dulger et al.	Turkey/2011	37/F	Pain abdomen, distention and hypotension. CECT suggestive of splenic rupture and hemoperitoneum (1.5 L).	Peritoneal lavage and multiple blood transfusions. Combination anti-Brucella therapy for six weeks. Recovered fully.
3	Demirdal et al.	Turkey/2011	65/M	Fever, malaise, headache, anorexia, thrombocytopenia.	Conservative management, anti-Brucella therapy for six weeks, and multiple platelet transfusions.
4	Leon et al.	Spain/1990	N/A	N/A	N/A
5	Rivera et al.	Spain/1982	N/A	N/A	N/A

The underlying mechanism contributing to spontaneous rupture of the spleen has been attributed to splenomegaly, especially pathological infiltration of the capsule, splenic infarct with subcapsular hemorrhage (subsequent rupture), and sometimes associated coagulopathy. However, rupture is known to occur from a combination of these mechanisms [11,14]. As postulated by Demirdal et al., the splenic capsule is thin and fragile. In addition, infectious processes might lead to congestion and dilatation in the sinusoids and Billroth cords, making the spleen susceptible to hemorrhage and rupture. Presumably, similar mechanisms may have been responsible in our case [11].

The typical presentation involves left upper quadrant (LUQ) pain, vomiting, abdominal distention, tenderness, and dizziness that may occur if hypotension develops. Kehr sign (referred pain to left shoulder) and Ballance sign (palpable tender mass in the left upper quadrant) may be present, as seen in our case where a tender palpable mass in LUQ was present [15].

Radiological evaluation via ultrasonography forms the mainstay in diagnosis, revealing an enlarged spleen, areas of decreased echogenicity, subcapsular and pericapsular hematomas, and the presence of intraperitoneal free fluid. Computed tomography (CT) having a higher sensitivity and specificity helps in the diagnosis and grading of splenic injury [16,17].

The treatment decision depends on a case-to-case basis, upon the grade of splenic injury, and the hemodynamic stability of the patient, as there is no clear consensus on treatment. In the presence of certain red flags like hemodynamic instability at presentation, high-grade splenic injury, precipitous fall in hemoglobin to less than 10 g/dL, or acute worsening of clinical condition (worsening abdominal pain, distention, and hypotension), a splenectomy along with exploratory laparotomy for draining of hemoperitoneum can be considered the procedure of choice [6]. However, in low-grade splenic injuries and hemodynamic stability with no further clinical worsening, conservative management is preferred to prevent unnecessary post-splenectomy complications [11,18]. Table 2 depicts the management and outcome of the previously reported cases, splenectomy was performed in one case while in the other peritoneal lavage was required. Both these cases had hemodynamic instability and widespread hemorrhagic fluid in the abdomen. In one of the cases, conservative management was done on similar lines to our case. Combination anti-Brucella management is common in all cases. Our case was managed conservatively and recovered completely with medical therapy. Although splenic rupture is an extremely rare complication of brucellosis, it should be kept as a differential if clinical presentation points to an acute abdomen.

## Conclusions

Spontaneous splenic rupture is a rare phenomenon and should be considered in patients presenting with acute abdomen and underlying suspected infective, neoplastic, and inflammatory diseases. Our case highlights the occurrence of spontaneous splenic rupture in acute brucellosis which requires prompt clinical recognition and immediate anti-Brucella therapy to prevent the catastrophic progression.

## References

[1] Zhen Q, Lu Y, Yuan X (2013). Asymptomatic brucellosis infection in humans: implications for diagnosis and prevention. Clin Microbiol Infect.

[2] Buzgan T, Karahocagil MK, Irmak H, Baran AI, Karsen H, Evirgen O, Akdeniz H (2010). Clinical manifestations and complications in 1028 cases of brucellosis: a retrospective evaluation and review of the literature. Int J Infect Dis.

[3] Monir MM (2001). Gastrointestinal brucellosis. Madkour's Brucellosis. Second Edition.

[4] Malik GM (1997). A clinical study of brucellosis in adults in the Asir region of southern Saudi Arabia. Am J Trop Med Hyg.

[5] Aubrey-Bassler FK, Sowers N (2012). 613 cases of splenic rupture without risk factors or previously diagnosed disease: a systematic review. BMC Emerg Med.

[6] Dumic I, Madrid C, Prada LR, Nordstrom CW, Taweesedt PT, Ramanan P (2020). Splenic complications of Babesia microti infection in humans: a systematic review. Can J Infect Dis Med Microbiol.

[7] Yağmurkaya O, Oğuz S, Kahya E, Aksoy H, Albayrak D, Sağıroğlu T (2021). Spleen rupture due to brucellosis. Turk J Surg.

[8] Dulger AC, Yilmaz M, Aytemiz E, Bartin K, Bulut MD, Kemik O, Sumer A (2011). Spontaneous splenic rupture and hemoperitoneum due to brucellosis infection: a case report. Van Tip Dergisi.

[9] Rivera JM, Pérez-Jiménez F, Rivera J, Mata M, Escauriaza J, Perepérez JA (1982). New case of spontaneous splenic rupture in brucellosis. [Article in Spanish]. Med Clin (Barc).

[10] León IM, González LM, Sillero AB, Parreño AM, Bendala CD, Bragado FG (1990). Atraumatic rupture of the spleen. A proposal of new attitudes. Apropos a case in brucellosis. [Article in Spanish]. An Med Intema.

[11] Demirdal T, Okur N, Demirturk N (2011). Spontaneous splenic rupture with hematoma in a patient with brucellosis. Chang Gung Med J.

[12] Renzulli P, Hostettler A, Schoepfer AM, Gloor B, Candinas D (2009). Systematic review of atraumatic splenic rupture. Br J Surg.

[13] Atkinson E (1874). Death from idiopathic rupture of the spleen. BMJ.

[14] Randriamarolahy A, Cucchi JM, Brunner P, Garnier G, Demarquay JF, Bruneton JN (2010). Two rare cases of spontaneous splenic rupture. Clin Imaging.

[15] Tu AS, Tran MH, Larsen CR (1997). Spontaneous splenic rupture: report of five cases and a review of the literature. Emerg Radiol.

[16] Putterman C, Lebensart P, Almog Y (1992). Sonographic diagnosis of spontaneous rupture of the spleen in infectious mononucleosis: case report and review of the literature. Isr J Med Sci.

[17] Jeffrey RB, Laing FC, Federle MP, Goodman PC (1981). Computed tomography of splenic trauma. Radiology.

[18] Stephenson JT, DuBois JJ (2007). Nonoperative management of spontaneous splenic rupture in infectious mononucleosis: a case report and review of the literature. Pediatrics.

